# Left hepatic vein injury during laparoscopic antireflux surgery for large para-oesophageal hiatus hernia

**DOI:** 10.4103/0972-9941.58501

**Published:** 2009

**Authors:** Anish P Nagpal, Harshad Soni, Sanjiv P Haribhakti

**Affiliations:** Department of Surgical Gastroenterology, Haribhakti Surgical Hospital, Ahmedabad, Gujarat - 380 006, India

**Keywords:** Complication, gastrooesophageal reflux disease, laparoscopic nissen fundoplication, left hepatic vein

## Abstract

Although the advent of laparoscopic fundoplication has increased both patient and physician acceptance of antireflux surgery, it has become apparent that the laparoscopic approach is associated with an increased risk of some complications and as well as the occurrence of new complications specific to this approach. One such complication occurred in our patient who had intra-operative left hepatic vein injury during laparoscopic floppy Nissen fundoplication for large para-oesophageal rolling hernia. With timely conversion to open procedure, the bleeding was controlled and the antireflux and the procedure were completed uneventfully. However, this suggests that even with an experience in advanced laparoscopy surgery, complications can occur. Clear understanding of the normal and pathologic anatomy and its variations facilitates laparoscopic surgery and should help the surgeon avoid complications. The incidence of some of these complications decreases as surgeons gain experience; however, new complications can arise due to the increase in such procedures.

## INTRODUCTION

Left hepatic vein injury during a laparoscopic fundoplication is rare. However, injury to blood vessels during this procedure can occur, such as bleeding from the short gastric vessels or the splenic vessels, aorta or hepatic veins. With an increase in the volume of laparoscopic surgeries, newer complications are also arising; however, with better understanding of the procedure these can be avoided or managed safely.

## CASE REPORT

**Figure 1:**
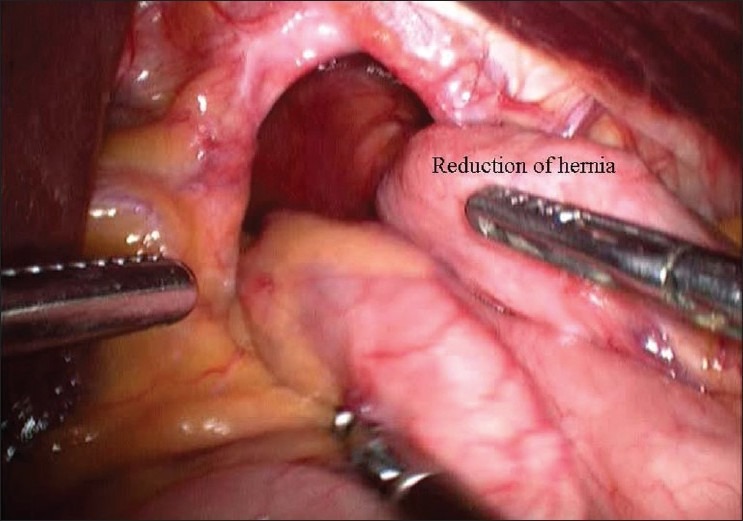
Intraoperative figure showing reduction of hiatus hernia

**Figure 2:**
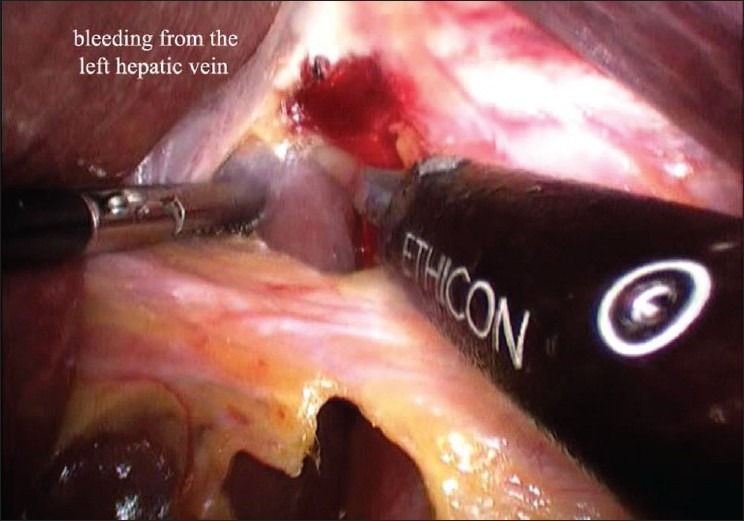
Bleeding from the left hepatic vein

**Figure 3:**
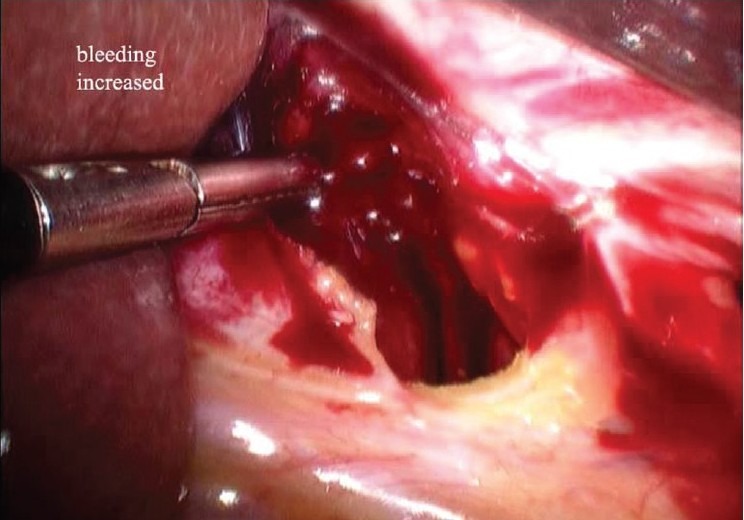
Bleeding uncontrolled laparoscopically

A 62-year-old male patient with no co-morbidities presented with complaints of fullness after meals since 3-4 months. Investigations were suggestive of large para-oesophageal hiatus hernia. Upper GI endoscopy was done which was suggestive of large para-oesophageal rolling type of hiatus hernia. A barium meal test was also done. The patient underwent a standard laparoscopic Nissen fundoplication for the repair of a hiatal hernia and correction of reflux. A Harmonic scalpel was used as the only energy source intra-operatively. The operation was commenced with the reduction of the large rolling hernia [Figure 1]. The lesser omentum (gastro hepatic ligament) was dissected. An aberrant left hepatic artery arising from the left gastric artery was encountered and secured. While dissecting to the right of GE junction, a small amount of bleeding was noted [Figure 2]. Injury to the left hepatic vein was suspected. After aspiration and careful inspection, a 1-2 mm vertical tear was found on the left hepatic vein. Attempt to control the bleeding from the tear was unsuccessful, as the bleeding became brisk [Figure 3]. The surgery was converted to an open procedure. At open surgery, the falciform ligament was divided and suprahepatic IVC was exposed. The left triangular ligament was divided and the left hepatic vein was exposed. Bleeding was controlled by suturing with 4-0 prolene. Later, the antireflux surgery was completed without further incident. The postoperative course was uneventful and the patient was discharged after 3 days.

## DISCUSSION

After comprehensive evaluation of this case to assess a potential cause of the complication, the question arose as to whether the anatomy was aberrant or the left hepatic vein anatomy was disturbed due to continuous and chronic traction from the larger para-oesophageal hernia.

Laparoscopic fundoplication has emerged as an effective and long-lasting therapy for gastrooesophageal reflux disease (GERD). Several studies[1–3] have shown that laparoscopy is a safe and effective procedure with good results. However, the laparoscopic procedure has its complications, and newer complications are encountered as the procedure volume increases. Some of these problems are unique to the laparoscopic approach, and others are more common following laparoscopic surgery.[4] Most complications are minimal, but they may be severe and even fatal.[5]

The greatest risk of the laparoscopic procedure is an unsuspected perforation of the oesophagus or stomach. An incidence of unsuspected bowel perforation in approximately 2% of cases is reported with the open procedure and has a high mortality.[6] Other potential complications include pneumothorax and injury to other organs. Bleeding from a vessel during this surgery is rare and if present is mostly from the gastrosplenic vessels. There have been reports of vascular injury to the inferior vena cava, the left hepatic vein, the abdominal aorta and the inferior phrenic vessels.[7,8] Laparoscopic fundoplication is an almost bloodless procedure, even when short gastric vessels are divided. When major haemorrhage does occur, it is imperative that the decision to convert to laparotomy is made promptly. Conversion and control of bleeding takes time and should be done before the patient becomes exsanguinated.

This injury was associated with aberrant anatomy. An extremely large (12 mm in diameter) aberrant left hepatic vein was probably never encountered before and its proximity to the hiatus made it even more vulnerable. Oesophageal injury is widely perceived to be the most serious complication of hiatal dissection.[9] Clearly, vascular injury may be life threatening, and the risks illustrated in this report can be minimized by (1) dissecting in the region of right crus and remaining medial to it in between the two crus; (2) realizing that the altered anatomy can be due to continuous traction in a case of larger rolling type of hiatus hernia. Early recognition of the aberrant anatomy may help in preventing a catastrophe and (3) early conversion to laparotomy, whenever brisk bleeding occurs which is not immediately controllable.
